# An evaluation of forensic casework in Greece: the impact of migration on the medicolegal system and a call for systematic decedent accounting for case resolution

**DOI:** 10.1007/s00414-026-03787-0

**Published:** 2026-04-14

**Authors:** Sophia R. Mavroudas, Despoina E. Flouri, Christina Karydi, Antonios Papadomanolakis, Elena F. Kranioti, Konstantinos Moraitis

**Affiliations:** 1 Forensic Anthropology Center at Texas State, Department of Anthropology, Texas State University, San Marcos, TX USA; 2 Forensic Medicine Unit, University of Crete, Heraklion, Crete Greece; 3 Department of Forensic Medicine and Toxicology, School of Medicine, National and Kapodistrian University of Athens, Athens, Greece; 4 Forensic Pathology Division of Crete, Hellenic Ministry of Justice, Transparency and Human Rights, Heraklion, Crete Greece

**Keywords:** Forensic anthropology, Unidentified human remains, Humanitarian forensic action, Long-term unidentified, Migration fatalities

## Abstract

This paper uses retrospective case data from across three forensic services in Greece to evaluate the process of identification for unidentified human remains recovered within Greece. The cases included in this study (*N* = 592) cover the majority of forensic anthropology cases for the country and all unidentified forensic pathology cases for the island of Crete. The retrospective analysis shows that the demographic data between migrants and non-migrants in the dataset are distinct and that in addition to migrant countries, the Greek medicolegal authorities deal with identifications across many continents due to high levels of tourism. The data also show that there is a large impact on the forensic services when migrant shipwrecks occur within the jurisdiction of these services. The absence of migrant decedent data in this data set compared to expected numbers shows a gap in accounting of migrant remains within the seas surrounding Greece. The data also shows that case resolution for unidentified human remains found within Greece is complicated by a lack of interagency collaboration and infrastructure despite current improvements to the medicolegal capacity of the country.

## Introduction

The silent mass disaster of unidentified human remains within medicolegal offices globally necessitates investigation of the process of identification to improve protocols and decrease the number of unidentified human remains languishing in offices across the world. This problem is exacerbated in areas of high migration where migrant deaths contribute to increases in casework with lack of corresponding funding. According to the International Organization for Migration (IOM)’s Missing Migrant Project, these high-migrant-traffic areas include the Americas, Europe, the Mediterranean, Africa, Western Asia, and Asia with reported numbers of missing migrants in these areas ranging from one thousand up to almost thirty thousand at the time this article was written [1].

For unidentified human remains found in these liminal spaces, it is necessary for forensic practitioners to search for corresponding missing persons data for identification. The location of this missing persons’ data is dependent on whether the individuals are nationals of the country in which they are found, temporary visitors, or migrants (both registered and unregistered) from external countries. This issue can be further complicated in touristic areas, like in Southern Europe, where deceased tourists can overburden medicolegal offices during peak tourist seasons. The estimation of population affinity and establishment of missing persons databases within forensic casework is crucial in these regions to direct the initial search for antemortem comparative data. The challenges with estimating population affinity in border zones, however, are great and have been discussed in depth by anthropologists working in these areas in both the US and Europe [2].

In the US in particular, the ever-expanding range of countries-of-origin for migrants entering the US also places increasing burdens on the medicolegal systems’ attempt to identify deceased migrants in their jurisdictions. As an example, the establishment of the National Missing and Unidentified Persons System (NamUs) in the United States has provided an opportunity for jurisdictions to cross-reference unidentified human remains data with missing persons reports outside of their immediate jurisdictions. Along the US border zones, however, the presence of unidentified migrants forces investigators to look outside of NamUs for missing persons from foreign countries, primarily from Mexico and Central America, although increasingly the scope of countries from which migrants originate is expanding [3, 4]. Often, practitioners working in border zones in the United States cannot rely on NamUs for identifications but instead need to work with non-governmental organizations from several countries to gather missing persons data and family reference samples [5]. This situation is similar to the issues medicolegal professionals in Southern Europe face when attempting to identify migrants crossing both the Mediterranean Sea and the Greek-Turkish land border.

Without accessible, searchable databases, finding missing persons data from outside of the country in which someone is found is cumbersome not only because of potential communication roadblocks, but also because of a lack of antemortem data such as DNA family reference samples, fingerprints, medical records, or antemortem photographs. A recent review of the status of unidentified remains globally by Reid et al. [6] highlighted that many countries lack within-country missing persons databases, which prevents cross-country data searching in increasingly globalized areas. In areas impacted by high levels of migration, there aren’t publicly available databases like NamUs for cross-referencing on a national level, let alone transnationally. This gap in knowledge makes it hard to evaluate the impact of data sharing and/or database development on case resolution. As more deceased unidentified migrants from southeast Asia, the middle east, and Africa are being discovered along both the European and US Southern borders, having cross-contextual comparative data across these areas of the world would help inform policy and procedures regarding this tragic, silent mass disaster [2].

This paper is focused on the unidentified casework in Greece, a country at an important first arrival point of migration into Europe. Within Greece, the jurisdiction over investigation of death falls within three separate ministries namely: the Ministry of Justice, Transparency and Human Rights, the Ministry of Education, and the Ministry of Health [7, 8]. Each department is responsible for cases that are discovered within their geographical jurisdiction and have varying resources and types of expertise for case management, identification, and, ultimately, case resolution.

**Fig. 1:**
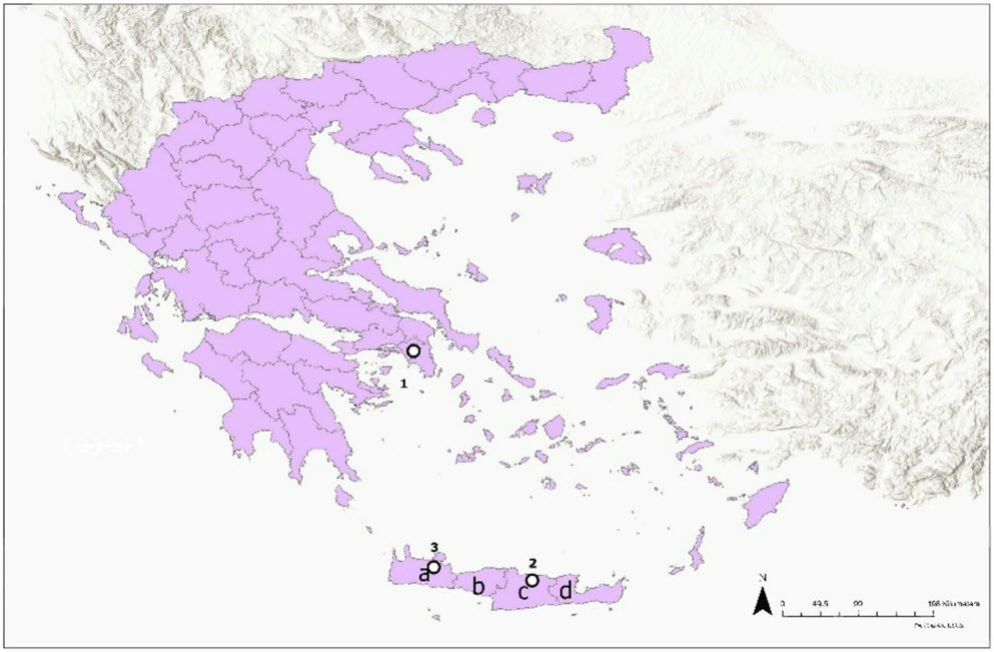
Map of Greece showing the locations (1–3) of each forensic service in this study including the Department of Forensic Medicine and Toxicology at the National and Kapodistrian University of Athens (NKUA), the Forensic Medicine Unit at the University of Crete (FMU), and the Forensic Pathology Division of Crete (FPD). The map outlines all regional units of Greece and labels the four prefectures of Crete including Chania (**a**), Rethymnon (**b**), Heraklion (**c**), and Lasithi (**d**)

The data for this study was collected retrospectively from three forensic services (Fig. 1). Two of the services are within the Ministry of Education (the Department of Forensic Medicine and Toxicology of the National and Kapodistrian University of Athens and the Forensic Medicine Unit of the University of Crete), and one within the Ministry of Justice, Transparency, and Human Rights (the Forensic Pathology Division of Crete).

### Department of forensic medicine and toxicology of the National and Kapodistrian University of Athens (NKUA)

The casefiles from Athens used in this study pertain to forensic anthropology casework received from all prefectures of Greece. In NKUA cases, a forensic anthropology report is written by the anthropologist and may be a stand-alone report or sometimes attached to the autopsy report from the same department. Since this is the only institution in the country with an established Forensic Anthropology Unit [9], the department receives cases from investigative authorities across the mainland and from the hundreds of islands in the country, including some Ministry of Justice cases from the western portion of Crete and some mass casualty events (shipwrecks) from central Greece and the Peloponnese. Considering these are anthropology cases, many of the individuals are discovered in advanced stages of decomposition [10] or even skeletonization, which can greatly reduce the numbers of pathways in which an identification can be made, specifically prohibiting visual identification and fingerprinting. Although the department maintains a custom-built digital database, known as the Athena Forensic Information System (AFIS), for managing forensic pathology cases, forensic anthropology cases are not yet integrated. As a result of these circumstances, data from this service was collected from a combination of digital records and hard copy casefiles.

### Forensic medicine unit of the University of Crete (FMU)

The casefiles used in this study are cases of forensic pathology and forensic anthropology reports that come primarily from the eastern side of the island of Crete, from the prefectures of Heraklion and Lasithi (Fig. 1). Although there is no officially established Forensic Anthropology Unit at the University of Crete, there is a dedicated forensic anthropology lab as part of the Forensic Medicine Unit, with a Level I Forensic Anthropology Society of Europe Certified Forensic Anthropologist. Since 2024, forensic specialists from FMU form part of the Greek Disaster Victim Identification Unit. The nature of this department means that the casefiles are from more varied states of decomposition when compared to the NKUA cases, thus exhibiting more potential avenues for identification, such as fingerprints and visual identification. This department has a custom-made digital database known as IPPASOS [11] for case management and documentation of long-term unidentified cases. A special feature developed within IPPASOS is the automatic inclusion of any case logged in as “Unidentified” in an automatically renewable database of unknown remains, which can be extracted by the users and shared with pertinent law enforcement departments for cross – referencing.

This department also utilizes postmortem CT scans for casework and has archived postmortem CT scans available for the long-term unidentified remains from 2016 onwards. The data from this service was collected through queries of the IPPASOS database.

### Forensic pathology division of Crete (FPD)

The casefiles from the Ministry of Justice, Transparency, and Human Right’s Forensic Pathology Division in Crete are cases of forensic pathology reports that come primarily from the western side of the island of Crete, from the prefectures of Rethymnon and Chania (Fig. 1), with some older cases dating prior to the year 2000 originating from the eastern provinces. The nature of this department means that the casefiles reflect individuals found in various stages of decomposition similar to the FMU casefiles, with avenues for identification that include visual identification, dental identification, fingerprints, and DNA analysis. This department has no digital case file documentation and no centralized database; thus, the physical archive was accessed for data collection. Data collection from this division was complicated due to the analog nature of the paper filing system and the lack of a uniform case numbering system.

Across all of these services (NKUA, FMU, FDP), there are three subcategories of cases, each necessitating a different pathway for investigation of missing person’s data. The first category is that of Greek nationals or permanent foreign residents, individuals who live in Greece and whose missing person’s data is likely within Greece. For reference of size, current estimates of Greece’s population are 10,361,295 individuals according to the most recent data from the World Bank [12]. The second category is that of migrants, individuals who travel from their country of origin either to live and work in Greece or are attempting to travel through Greece to enter Europe. The numbers of migrants entering Greece vary. At the height of migration in 2015, the IOM estimates 857,363 migrants arrived by both land and sea into Greece [13]. Since 2015, there has been a steady flow of migration to Europe, primarily through sea routes across the Mediterranean and land routes along the Greek and Balkan borders. Current estimates of the number of migrants entering Greece suggests much smaller numbers since 2015, but still substantial numbers of cases averaging about 40 thousand migrants arriving in Greece each year over the last three years [14]. The full scale of the phenomenon is considered to be grossly underestimated, especially on account of low recovery rates of deceased migrants from marine settings. The third category of cases is that of tourists, individuals who travel to Greece for tourism and end up missing while in the country. At the same time, tourism accounts for above 19% of Greece’s gross domestic product and the estimated number of tourists entering Greece in 2023 was over 36 million, a record high and a number that surpasses that of the country’s local population [15] by almost three and half times.

When examining the approach of identification methods for each of these categories (residents/migrants/tourists), it is likely that repositories of missing persons data for identification is managed by differing entities for each category of unidentified person, although there has been little systematic data collection into identification methods in Greece to make solid conclusions. The task of identification in Greece is legally attributed to law enforcement, while forensic practitioners collect evidence from the human remains and are typically not involved in the task of identification but rather informed of it. From anecdotal data and daily practice, it is likely the missing persons data for Greek nationals is only within the jurisdiction in which a person went missing rather than in a widely accessible national database. While there is a newly developed centralized database for missing persons in Greece, run by the Missing Persons Bureau of the Hellenic Police (personal communication), there is no guarantee that missing persons case information has been passed to this centralized database. In the experience of the authors of this paper, there is a wide chasm between the data that individual jurisdictions possess and the information that is curated in the centralized database. As a result, “looking at the bigger picture” when trying to search for potential missing person matches across Greece is not always a viable option for forensic practitioners, or even investigative authorities such as the police or coast guard.

An example of this problem is reflected in the lack of resolution of an unidentified person’s case from 2012 from the FMU. The individual was found partially skeletonized inside an abandoned building, without any identification or other personal documents. The forensic anthropology report presented a biological profile and several identifiers amongst which was a surgical plate with a serial number. The FMU managed to trace the surgical plate back to the manufacturing company and thus led to the generation of a list with 20 potential patient matches (whose personal information can only be shared through a court order due to doctor – patient confidentiality clauses) from 5 European Hospitals all outside of Greece. Twelve years later, and despite the work of the FMU, including DNA samples and a full biological profile, no productive cooperation between INTERPOL and Greek authorities was achieved. It is evident that the existing forensic infrastructure between local, national and international authorities and forensic services is lacking which contributes greatly to the ongoing problem of unidentified deceased individuals.

The aim of this study specifically is to document the status of unidentified individuals recovered in Greece from these three services for the years 1988–2023 in order to (1) evaluate the process of identification across all categories of missing persons, (2) analyze demographic trends of forensic casework within the country, and (3) provide baseline data to direct future data collection efforts aimed at improving identification rates and infrastructure. Ultimately, this study aims to contribute to research that can help inform policy on the silent mass disaster of unidentified persons globally.

## Materials and methods

From each of the casefiles across the services of the NKUA, the FMU, and the FPD, the following demographic data was collected when available: estimated/known sex, estimated/known age, cause of death, manner of death, location and date of recovery, jurisdiction of the case, migrant status, identification status, and method of identification. If the case files commented on the availability of photographs of the deceased and associated personal items, postmortem imaging data or other physical and digital evidence, the existence of this data was recorded. All cases that were initially unidentified when received by the forensic service were included in this dataset. All of the data collected for this study has been included in a shared database to facilitate reanimated attempts at positive identification when possible.

Estimated sex was recorded from case reports and often estimated from either soft-tissue, anthropological analysis, or DNA results. Estimated age was recorded if stated in the pathology or anthropology report.

Cause and manner of death were recorded when available from pathology reports. Location of recovery and date of recovery was recorded from police inquiry reports when available and associated with the pathology/anthropology reports. If no exact location of recovery was available, the location of the police jurisdiction was used in GIS analysis. In cases of identified remains for which there was a corresponding missing person’s report recovered in the police file, data from that report were also collected.

In order to address the primary aim of this paper, which is to understand the unidentified forensic population in Greek medicolegal casework, the nationality of all identified individuals was recorded when available along with migrant status. After data collection was complete, it was possible to group the cases into two main groups: migrants and non-migrants. Due to the difficulties associated with assigning migrant status to unidentified individuals [16] migrant status for this study was only considered positive if a positive identification was made. The exception to this was if an individual was recovered from a known migration-related fatal incident. In these cases, the individuals were grouped with the migrants despite the absence of a positive identification. This category primarily consists of unregistered migrants attempting to enter the country through unofficial routes and channels. The non-migrant group consists of the following subgroups: Greek nationals, long-term residents of Greece, and tourists. For some analyses, Greek nationals were further subdivided from the non-migrant group as indicated in the results.

Because of the fragmented nature of the case files and differential data collection not all information was available for every case. In the results below, all summary statistics are presented from all information that was available. Any missing statistical data is due to lack of available information in the casefiles, including demographics and identification methods.

All maps were made using ArcGIS Pro 3.3.0. The case recovery locations are presented as both individual geolocations and in density analysis. Standard density tools were incorporated.

## Results

**Fig. 2:**
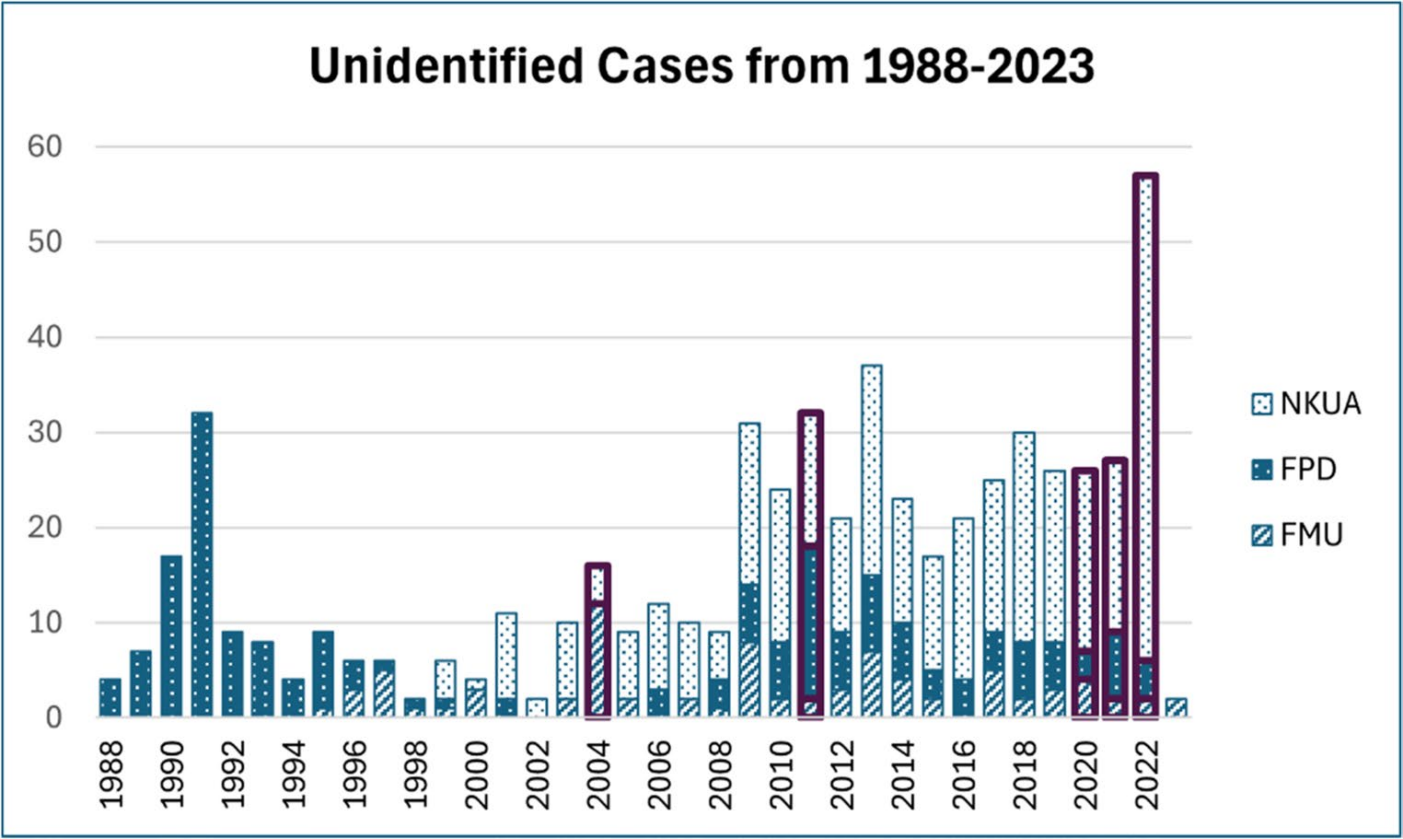
Overall distribution of cases labeled by investigative agency including the National and Kapodistrian University of Athens (NKUA), the Forensic Pathology Division of Crete (FPD), and the Forensic Medicine Unit (FMU) of the University of Crete. Years outlined in bold indicate years in which migrant remains were recovered including 2004, 2011, 2020, 2021, and 2022

Between the years 1988 and 2023 inclusive across these three services, a total of 592 forensically significant cases of unidentified human remains were collected (Fig. 2). Of this number, 342 individuals were recorded as identified (57.7%). Of the 342 identified individuals, 178 were Greek nationals and 32 were migrants. The remainder of the identified persons were long-term residents/tourists (*n* = 132). An additional 25 migrants were unidentified but marked as known migrants because they were recovered from documented migration-related mass fatality incidents (migrant shipwrecks).

### Demographic data

**Fig. 3:**
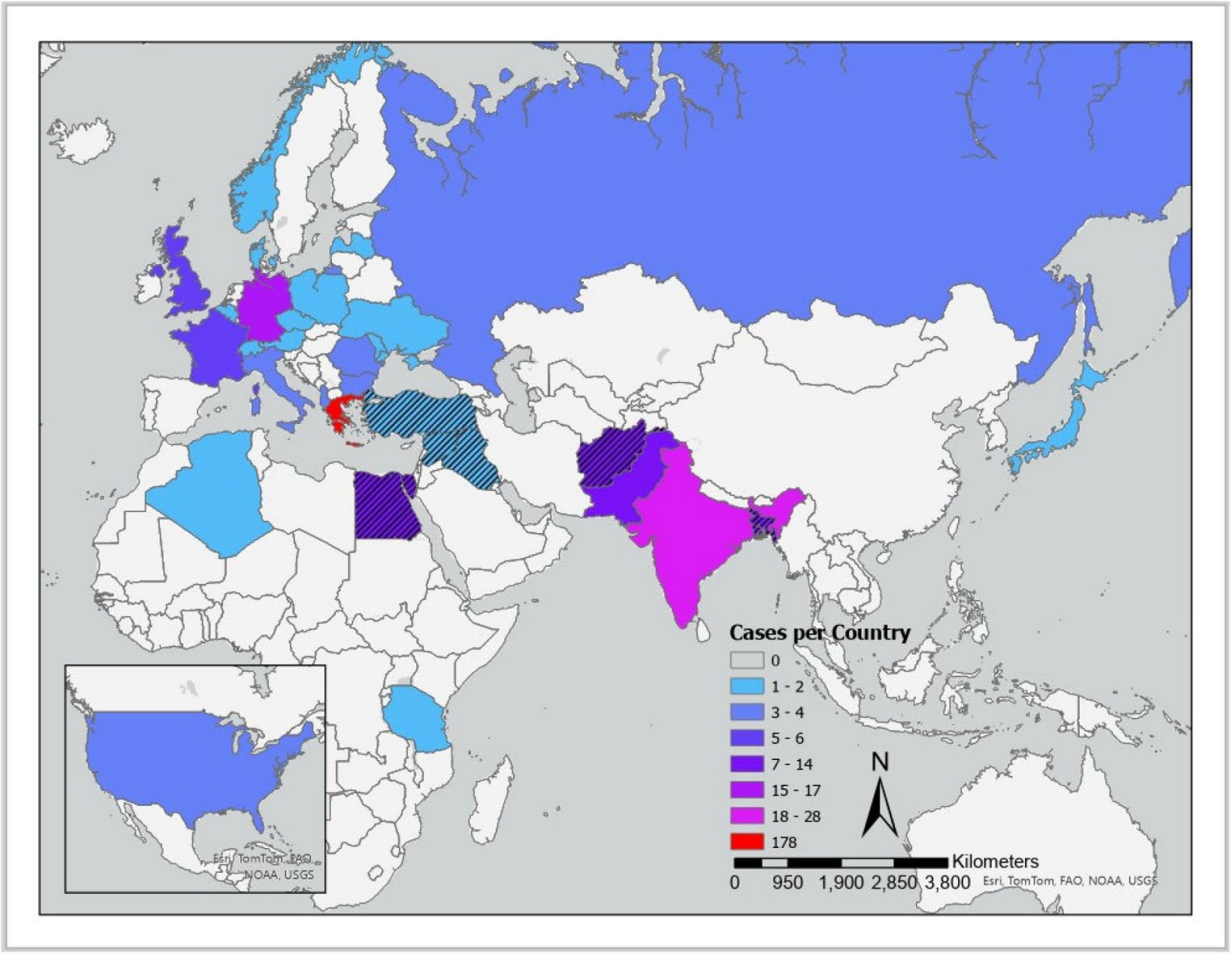
Map of the world with countries of origin for all identified cases. The legend shows the scale of the number of cases per country. The inset area shows North American cases. Hashed countries indicate countries of origin for the migrant cohort of the dataset

Out of the 592 cases, 67 per cent of them were male and 18 per cent were female, the remaining had no documented sex assessment. A lack of sex assessment was often the result of incomplete casefiles and/or of cases consisting of isolated skeletal remains/elements. The overall age range for known-age individuals was 6–94 years with a mean age of 52.3 years. For all cases, the identified individuals originate from a total of 32 countries including Afghanistan, Albania, Algeria, Austria, Bangladesh, Belgium, Bulgaria, Czech Republic, Denmark, Egypt, France, Georgia, Germany, India, Iraq, Italy, Japan, Korea, Latvia, Moldova, Norway, Pakistan, Poland, Romania, Russia, Switzerland, Syria, Tanzania, Turkey, Ukraine, the United Kingdom, and the United States (Fig. 3).

When the data is broken down further by sex, the demographic profile changes slightly. The age range for males with known ages is 6–94 years with a mean age of 51 years. The age range for females with known ages is 6–93 years with a mean age of 57.6 years. For all cases, irrespective of the identification status, each individual was assigned to an age group based on either a known-age or an estimated age from anthropology or pathology reports. These groups were (i) under 18 years of age, (ii) 18–60 years of age, or (iii) over 60 years of age. Most of the cases fell into the second group. When divided by sex, the male group had more individuals under the age of 18 (*n* = 14) than the female group (*n* = 5).

A total of 58 per cent of cases were identified across all services. When the identified individuals (*n* = 342) are broken into the subcategories of migrant vs. non-migrant as described earlier, the demographic data trends shift (Table 1). For the identified non-migrant group 75% are males with ages ranging from 7 to 94 years with a mean age of 55.5 years. Another 22 per cent are females with ages ranging from 6 to 93 years with a mean age of 59.7 years. There were some cases marked identified, with no demographic information for sex or age which explains the missing data in this identified category. The identified non-migrant individuals exhibit documented nationalities from twenty-eight countries including Albania, Algeria, Austria, Belgium, Bulgaria, Czech Republic, Denmark, Egypt, France, Georgia, Germany, Greece, Italy, India, Japan, Korea, Latvia, Moldova, Norway, Pakistan, Poland, Romania, Russia, Switzerland, Tanzania, the UK, Ukraine, and the US. Greek nationals can be further subdivided from the non-migrant group as seen in Table 1. This subgroup has a higher mean age than the other groups and larger percentage of natural deaths.

For the confirmed migrant group (*n* = 57) the demographic trends show 86 per cent are males with an age range of 6–60 years and a mean age of 26.5 years for those with known ages (Table 1). The migrant females make up 14 per cent of the group with an age range of 11–60 years and a mean age of 30.2 years for those with known ages. The migrant group have documented nationalities from six countries including Afghanistan, Bangladesh, Egypt, Iraq, Syria, and Turkey. The country with the highest frequency of migrants represented in this data set is Bangladesh and Egypt with 13 individuals each followed by Afghanistan with 11 individuals. The migrants from Bangladesh died in a shipwreck in 2011 off of the western coast of Crete. The migrants from Egypt died in 2022 in a shipwreck off the coast of Central Greece. The migrants identified from Afghanistan were recovered from two different shipwrecks, the first in 2020 off the coast of Crete, and the second in 2022 along the coast of Central Greece. There was a total of *n* = 25 migrants with no known identity but each of them had known/estimated sex and were included in this data. Interestingly, the composition of the countries of origin in this cohort differ to cases received by the Forensic Services of the Southern Aegean Islands (personal communication), in which the predominant nationalities included Iraq and Syria. This is an observation that can be attributed to different migration routes.

**Table 1:**
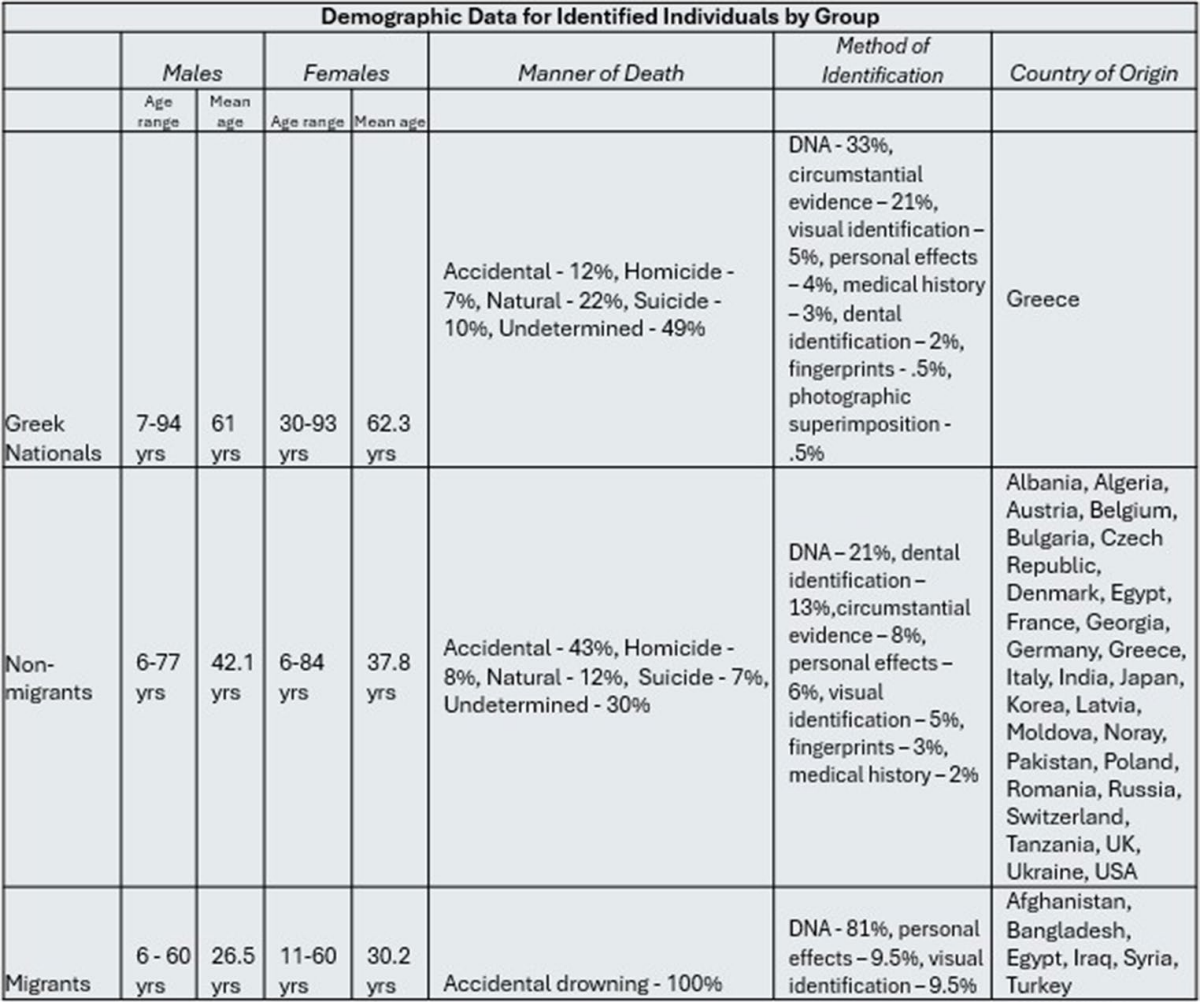
Table showing data demographics, manner of death, methods of identification and country of origin subdivided into groups: Greek Nationals, Non-migrants (excluding Greek nationals), and Migrants

### Cause and manner of death

The non-migrant group had “accident” as the most commonly identified manner of death, with 52 per cent of the accidents being drownings. Additional accidental causes included “falls from height” and traffic-related deaths. The non-migrant group also included natural, suicidal, and homicidal manners of death. The only manner of death recorded for the migrant group was accidental, with the cause being drowning in sea waters (Table 1).

### Methods for identification

When looking at methods for identification across the entire dataset, most of the cases with documented identification methods (*n* = 231) were identified using DNA (*n* = 113). The other means of identification were recorded as documented in the casefiles and included circumstantial information (*n* = 46), dental records (*n* = 20), personal effects (*n* = 19), visual identification (*n* = 19), medical records (*n* = 8), fingerprints (*n* = 5), and photographic craniofacial superimposition (*n* = 1) (Table 1). Not all visual identifications were confirmed by a relative. At least one documented case of visual identification by a priest was recorded, and often the files simply indicated that a visual identification was performed, with no clarification as to the identifying party. The photographic superimposition was done through a partial overlay of the maxillary area of the craniofacial skeleton. Although this information was not in the casefile, it was confirmed through discussion with a practitioner working at the laboratory at the time. Many casefiles had no information on method of identification (*n* = 105).

When looking at identification methods separated by sub-group, the non-migrant group had a greater diversity of identification methods including DNA for 40% of the individuals, but also fingerprints, visual identifications, personal effects, circumstantial information (e.g. recovered near last known location), and the single record of photographic superimposition. Further dividing the non-migrants, the Greek nationals were identified primarily through DNA (33%), followed by circumstantial evidence (21%), but also included dental identification, visual identification, personal effects, medical history, one case was identified through fingerprints, and one through photographic superimposition. The primary method of identification for migrants was DNA analysis (81%) but also included personal effects and visual identification by a relative (Table 1).

### Geographic distribution

**Fig. 4:**
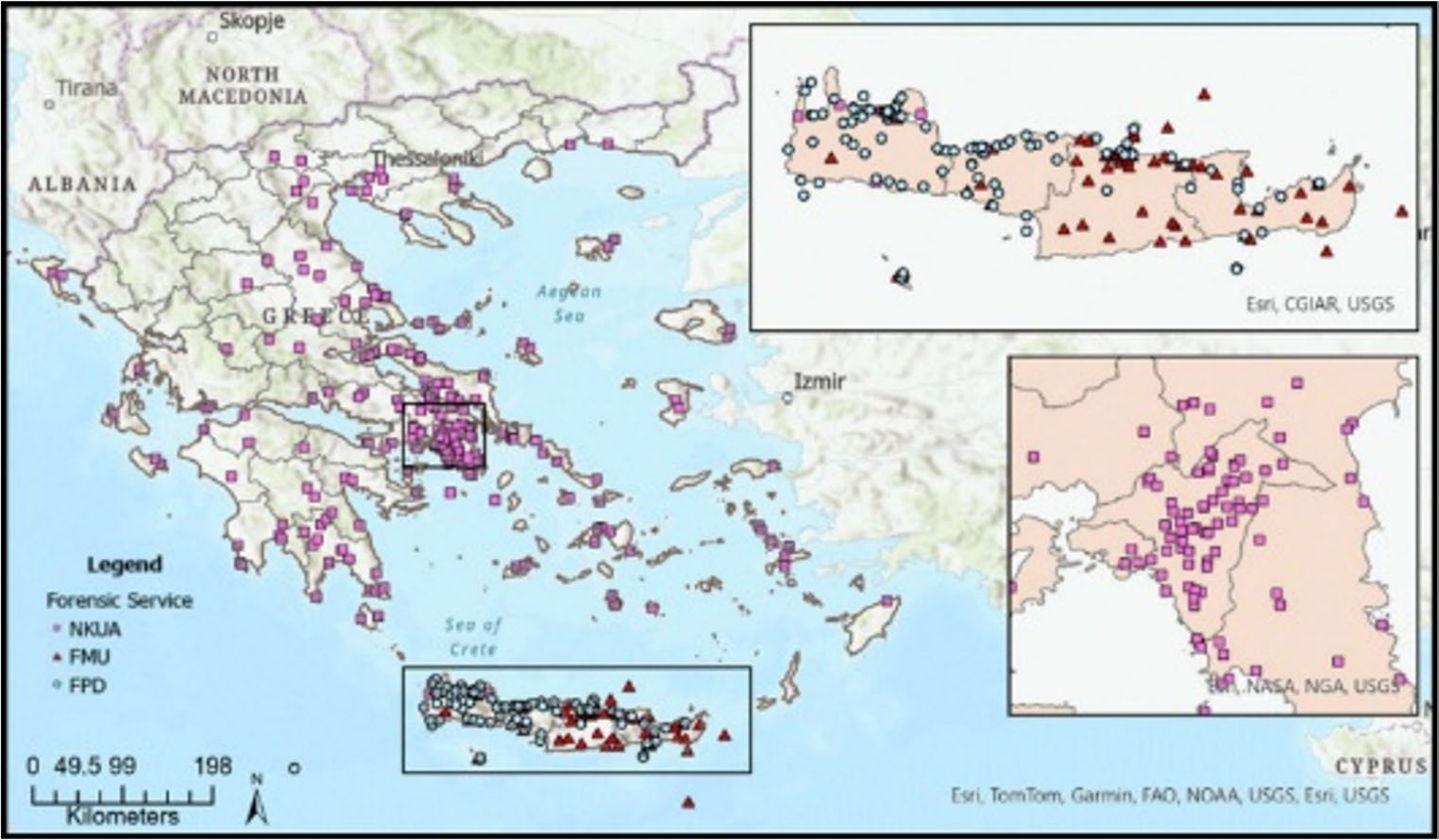
Geographic distribution of cases across Greece labeled by case service. Insets show close-ups of cases in and near Athens as well as on Crete categorized by service. The majority of the Cretan cases are along the northern border of the island which is consistent with population density of the island

**Fig. 5:**
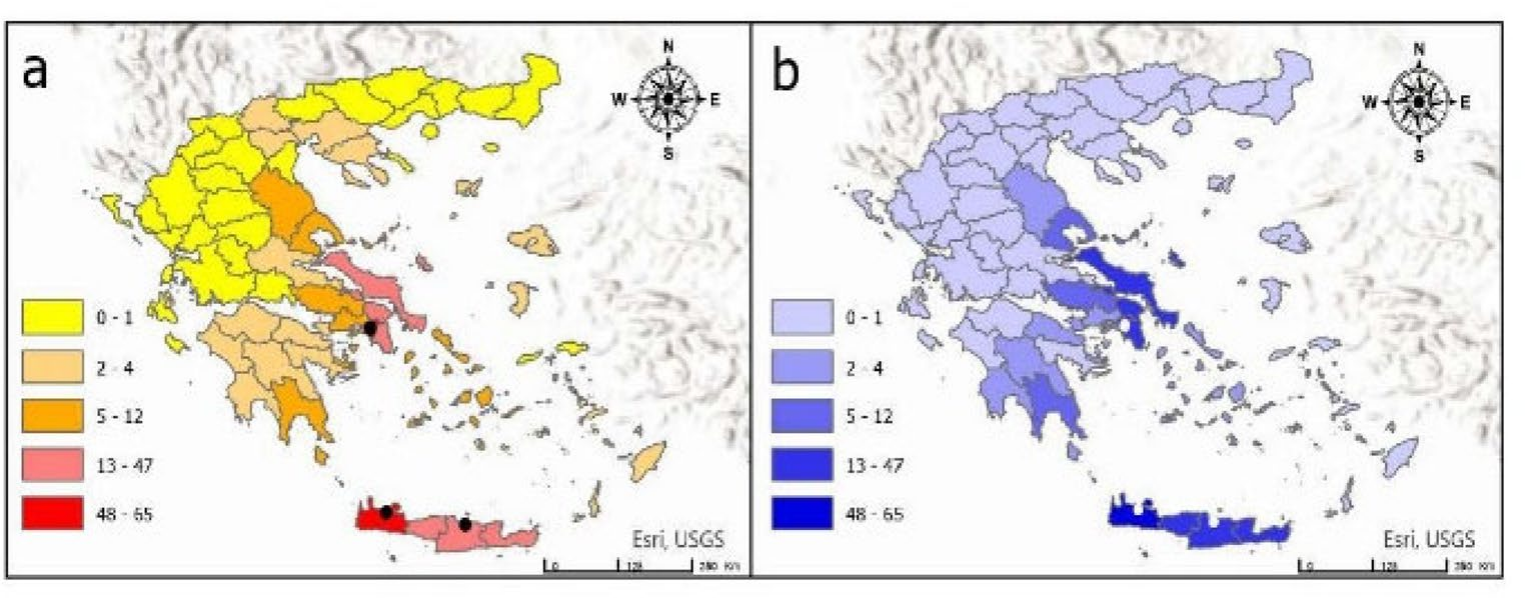
Maps of Greece showing the number of cases recorded per region by recovery location. Map a showing the total number of cases recovered within each prefecture and Map b showing the total number of cases that were identified within each prefecture. The number of identifications per region corresponds to the regions with the highest case numbers as expected, but Map b also shows that proximity to service city (white circles) corresponds to increases in identification rates. Cases at sea are not included within regions

**Fig. 6:**
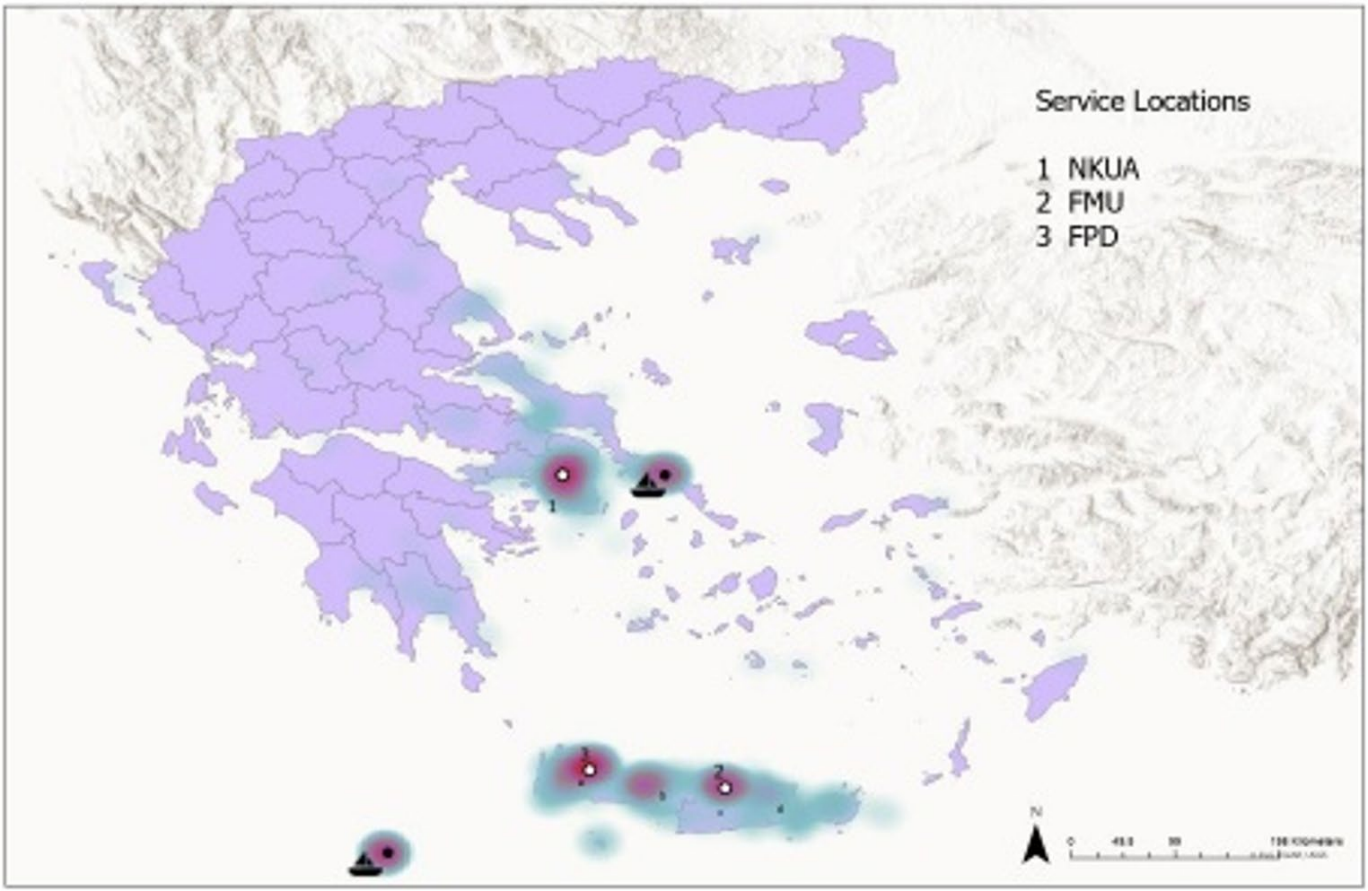
Map of Greece showing density plots of identified cases from the entire dataset. The analysis shows concentrations of identified individuals centered around the city of the forensic service in Athens and the larger cities in Crete (Chania, Rethymnon, and Heraklion). This hotspot map also shows increased identification rates for two contained mass-disaster shipwrecks, one known migrant shipwreck to the east of Athens and one non-migrant shipwreck to the west of Crete

Looking at the spatial distribution of cases by individual locations (Fig. 4) and by prefecture/region (Fig. 5), it is obvious that NKUA accepts cases which cover the majority of the regions within Greece, while the FMU and the FPD focus on the island of Crete and the surrounding sea spaces. Despite this coverage, the map also shows the major concentration of cases near the department cities: Athens, Heraklion, and Chania respectively. Looking at hotspot analysis of the identified individuals (Fig. 6) irrespective of region in this dataset it is evident that identification is more likely for individuals found closer to major cities near the department locations specifically, Athens, Heraklion, Rethymnon, and Chania. The hotspot map also reflects a large number of identifications for two separate shipwreck incidents. One pertains to a migrant shipwreck to the east of Athens handled by the NKUA with many individuals identified through DNA analysis [17], and the second to a shipwreck that involved a cargo ship from India which occurred to the west of the island of Crete handled by the FPD with undocumented methods of identification.

## Discussion

The analysis of this data shows that most unidentified individuals across these three offices were non-migrants, and that the primary recorded means of identification for unidentified individuals in these services was through DNA. This data also shows that Greek nationals only make up 30% of the identified individuals, which is significant to medicolegal practitioners and law enforcement agencies working in these Greek systems.

The high number of countries-of-origin for the cases represented in this dataset (*n* = 32) across multiple continents highlights how much procedure and identification processes can vary from case to case even within departments. The limited number of countries from which the migrants in this dataset were identified was surprising when compared to migrant data from other parts of Greece, specifically, the Greek-Turkish land border [18, 19]. Along the Greek-Turkish border, previous studies have outlined eighteen separate countries of origin for migrants crossing that land border [18]. This discrepancy is possibly due to the high number of shipwreck events from a single point of origin represented in this dataset as opposed to individual migrant deaths along the land border. At the same time, marine migration routes into Greece originate either from the Turkish Coastline, arriving onto the Southern and Northern Aegean Islands, or from Libya, arriving onto the Southern shores of Crete. Even though the Libya-Greece migration route is becoming increasingly popular, the number of mass casualty events is still small. On the other hand, the Southern (e.g. Rhodes) and Northern Aegean (e.g. Lesvos) islands have consistently been the main recipients of migration influxes, including fatal incidents. Those forensic cases have been handled by the Forensic Pathology Division of Dodecanese and the Forensic Pathology Division of the Northern Aegean and are not included in this present study. It is likely that as migration routes change, countries-of-origin for migrants processed through these services might change as well.

When looking at Table 1, the data shows an obvious difference in demographic trends between migrants and non-migrants in this dataset. Not only do the migrants make up a small percentage of the overall caseload, but within the migrant group, females make up a very small percentage of cases. These results are consistent with other migrant forensic data from Greece [17, 18] and other global border zones including the US [4], which shows primarily younger males making up the majority of the migrant forensic population who are perishing at the land borders.

The results of this study also show that DNA methods were the primary means of identification for both the migrant and non-migrant dataset, which was surprising, since the initial hypothesis was that the DNA for migrant cases would be difficult to obtain from their respective countries of origin. The use of DNA in migrant cases shows that established protocols for identification are successful when migrant remains are recovered from a known mass fatality incident. Table 1 also highlights how few avenues there are for migrant identification if there is not an identification hypothesis or a mass fatality incident. Compared to other areas of Greece with published information on methods of identification for migrant remains [18, 19], this dataset lacks any identifications of migrant remains based on fingerprints which show possible regional differences within Greece regarding migrant identification. One possible explanation is the fact that individuals attempting to cross land borders effectively travel through multiple countries and may have been flagged by the authorities as criminal offenders and fingerprinted, whereas sea arrivals directly reach the shores of Greece. Another possibility is that the migrant recovery locations reflected in this dataset (primarily in the sea or near the shore) impacts the ability for identification using fingerprints.

The shipwreck incidents in this dataset are a good reminder that large-scale shipwrecks are often distinct, high-profile events that receive significant attention from mass media. As a result, the families of individuals who were aboard these ships are often more aware of the likelihood that their relatives have perished, which increases the possibility of supplying antemortem data and DNA family reference samples. In contrast, isolated incidents of migrant deaths, which frequently go unreported or remain unnoticed, are less likely to be associated with coordinated identification efforts. Often, individuals travel with their families or friends and therefore survivors may be present to attest to the identity of the deceased. This can be seen in the data from this study where all migrants were identified from shipwrecks, and there were no isolated migrants identified in this sample. Although not marked as migrants, many individuals in this dataset were recovered in isolation from the sea or near the shore but without a country-of-origin hypothesis and nowhere to gather comparative antemortem data from, an identification could not be made. Until case protocols can include some hypothesis about country-of-origin (either through personal effects, forensic genetic genealogy, or anthropological population affinity estimates) these cases are difficult to identify. Current use of population affinity estimation from skeletal remains is limited in these services, primarily because there is no relevant skeletal data to reference. This lack of comparative skeletal data is a widely discussed issue in anthropology, especially in border zones [2] and the need to collect relevant skeletal data is supported by this study.

In looking at the geographic locations of recoveries in this dataset it is also glaringly obvious that there is a lack of cases recovered within the sea. The absence of these cases in the dataset is similar to an absence of cases in a migrant dataset from Texas in the United States that researchers refer to as necrosilence [21]. Necrosilence, as discussed by Miranker and Giordano [21], occurs in areas where there is a lack of accounting of death incidences most likely related to a lack of access to the information. Through visual representation of the locations of recovery as in Fig. 4 of this Greek data, the necrosilences become visible in the sea in the same way they are visible in the desert in south Texas, in the US. These necrosilences indicate that there are unknown protocols for which human remains from these undocumented migrant shipwrecks are processed by local investigative authorities and smaller forensic departments. It would be helpful to practitioners to know how these cases are handled and identified, to help document how many deaths are occurring in this region and ultimately to increase identification rates across Greece and other high migration areas in the Mediterranean. The development of a centralized database for migrant fatalities, including shipwreck fatalities, might help with identification since these cases are often handled by varying forensic services across Greece and across the various Greek Ministries.

## Limitations

This study was dependent on the record keeping of the services in which these cases were performed and thus limited by the quality of the filing system. For the FMU, the data was easily accessible and query-able via their digital record keeping platform and all data is considered complete. For the NKUA, the casefiles were in physical copies, but all of the associated material was labeled with a universal case number and corresponding case information was easy to accumulate. The authors of this paper are confident that the data for the FMU and the NKUA encompasses all unidentified remains and associated data up through 2023. For the FPD, however, the case files were not digitally organized and had no universal case number for all associated case information. Since these files were organized by year and not cases, this meant that in order to associate police inquiry reports with pathology reports and possibly subsequent identification notices, every piece of paper had to be analyzed, and the analyst had to recognize when the reports corresponded to unidentified cases. This means that there is the potential that some identifications were not recorded in this dataset, although every effort was made to collect all available data.

A major limitation of this study is also that there are known unidentified cases (primarily of migrants) along the eastern border of Greece among the Northern Aegean islands that are not included in this dataset. Although a formal request for data has been made, there is no official accounting of migrant deaths for those who die in shipwrecks and do not go through the three services in this study. A potential future avenue for collection of this data is through cemetery surveys in these islands, which would help to provide some data on a minimum number of deceased migrants for these regions. This approach has worked on the US southern border [4, 21] and the authors of this paper hope to implement this strategy if casefile information cannot be obtained.

## Conclusion

Overall, the data presented in this study shows that for Greek medicolegal practitioners, unidentified non-Greek individuals constitute a large percentage of casework. Additionally, across these three services, there are clear demographic differences between migrants and Greek nationals that present within medicolegal casework including average age, cause and manner of death, as well as recovery location.

This study also shows how few migrants are captured in the casework in these services in a restrictive number of years (Fig. 2), despite documented high levels of migration into Greece during the time period this casework was completed [1, 13]. The results of this study indicate a need for an accounting of migrant deaths within the seas surrounding Greece, especially along the eastern border, to gather accurate identification rates and to be able to evaluate the effectiveness of existing policies. Without an accounting of unidentified persons cases, it is difficult to understand how the processes of identification can be improved and how the migrant crisis impacts forensic case resolution within Greece and beyond [22].

Additionally, when concisely looking at the dataset, there is the clear presence of a gradually increasing cohort of cases pertaining to singular bodies recovered from the sea or washed ashore (Fig. 4). Those bodies are usually not associated with known migrant or other mass casualty events (shipwrecks), they are recovered in advanced stages of decomposition, and they have no associated personal items or other identifiers. The way forward with these cases is unclear as there are usually no assumptions on the origins of the individuals. Such cases exhibit a 0 per cent identification rate and eventually turn into long-term unidentified cases. The need for consistent, postmortem CT imaging of these cases is imperative in this type of casework, to immortalize and store data for future reference. In addition to being available for future attempts at identification, the CT scans could also be used to develop cranial and postcranial reference data as discussed by anthropologists in humanitarian forensic action projects [2]. In addition to postmortem CT, attempts have been made recently to introduce craniofacial approximation to the Greek medicolegal and investigative system to resolve these contextless cases. This work is being done by pathologists at the FMU with support from a European Cooperation in Science and Technology grant [23] in collaboration with forensic artists from the Face lab at Liverpool John Moores University and the Hellenic Police.

Most importantly, the results of this study align with previous research on the European Southern Border which indicate that capacity building for identification of unidentified human remains is necessary to improve communication and identification rates across the country and the region [2, 24]. Despite the efforts of Greek stakeholders to provide resources such as a centralized missing persons database and a national DNA laboratory, a lack of appropriate training for law enforcement and medicolegal professionals leads to mis/disuse of these resources and ineffectuality of these resources for rural and remote regions of Greece as visualized in this dataset (Fig. 5). In light of recent publications highlighting the importance of Humanitarian Forensic Action in border zones [2, 25], it is especially poignant to all forensic practitioners to ensure they publish relevant data on case load, case demographics, and processes of identification to ensure policy promotes justice in these crisis zones. Without accurate accounting of fatalities an effective policy cannot be implemented.
